# The improvement of insulin level after hydrogen-rich water therapy in streptozotocin-induced diabetic rats

**DOI:** 10.14202/vetworld.2022.182-187

**Published:** 2022-01-28

**Authors:** Ekowati Retnaningtyas, Budi Susatia, Siti Nur Arifah, Sri Rahayu Lestari

**Affiliations:** 1 Department of Nursing, Politeknik Kesehatan Kemenkes Malang, Jl. Besar Ijen No. 77C, Malang 65119, East Java, Indonesia; 2 Department of Biology, Faculty of Mathematics and Natural Sciences, Universitas Negeri Malang (State University of Malang), Jl. Semarang No. 5, Malang 65145, East Java, Indonesia.

**Keywords:** diabetes mellitus, hydrogen-rich water, insulin receptor, insulin, superoxide dismutase

## Abstract

**Background and Aim:**

Water plays a pivotal role in the body. Alteration of the fluid balance promotes metabolic disorder, thus leading to the development of various diseases, such as diabetes mellitus (DM). Hydrogen-rich water (HW) is recognized as a novel antioxidant. This study aimed to investigate the role of HW on insulin, insulin receptor (IRs), and superoxide dismutase (SOD) levels in streptozotocin (STZ)-induced diabetic rats.

**Materials and Methods:**

A total of 30 male Wistar rats were randomly divided into five groups: Normal (N), DM rats, DM+metformin (DM+Met, 45 mg/kg body weight [BW]), DM+Met+HW, and DM+HW. DM rats were induced by feeding them a high-fat diet for 30 days and then injecting with repeated low doses of STZ (35 mg/kg BW) intraperitoneally. Fresh HW was administered orally and *ad libitum* for 14 days. Insulin, IRs, and SOD were observed in each group.

**Results:**

HW therapy increased the level and expression of insulin and IRs. In addition, treatment with HW also elevated the SOD levels in the serum and liver. The study results indicated no significant differences between the administration of HW and metformin.

**Conclusion:**

HW has antioxidant activity in STZ-induced DM rats, increasing insulin, IRs, and SOD.

## Introduction

Water plays numerous pivotal roles in biological processes in the body, including maintaining physiological function (e.g., blood pressure, pH, and body temperature) [1], being a carrier of nutrients (e.g., glucose, sodium, and potassium) and waste products [1,2], and being a lubricant, reaction medium, and building material [2]. Water is a universal solvent, which makes it the main constituent of living things [3]. Adequate water consumption is essential for maintaining body health. Alteration of the fluid balance elevates arginine vasopressin as a key hormone regulating body fluid homeostasis. These conditions lead to the development of type 2 diabetes mellitus (T2DM) [4].

DM is a metabolic disorder manifested by a high level of blood fasting glucose due to deficiencies in insulin secretion, action, or both [5]. Insulin is a hormone secreted by b cells of islets of Langerhans, and it binds to specific cell-surface receptors, namely, insulin receptor (IR) [6]. IR is located in the peripheral tissues, such as the skeletal muscle, liver, and fat [6,7]. DM is also closely associated with elevated oxidative stress status, thus lowering the level of endogenous antioxidants, such as superoxide dismutase (SOD), catalase, and glutathione peroxidase (GPx) [8]. However, a high-fat diet (HFD) is associated with increased free fatty acids, especially low-density lipoprotein (LDL), in the bloodstream. A high level of LDL promotes oxidized LDL [9]. These mechanisms elevate the level of free radicals such as reactive oxygen species and reactive nitrogen species that may induce cell death and organ damage, respectively, especially in pancreatic cells under DM conditions. Furthermore, antioxidant therapies have been widely used for DM treatments [10]. Exogenous antioxidants play a role in eliminating all types of free radicals and help increase the activity of endogenous antioxidants [11].

In water, one oxygen atom is covalently bound with two hydrogen atoms. The water has a polar structure, indicating that the electrical charge of water molecules is unevenly distributed [3]. Hydrogen-rich water (HW) is recognized as a novel antioxidant due to its ability to selectively scavenge strong oxidants, such as hydroxyl radical [12,13]. The significant result between HW and plain water consumption indicates that HW reduces the inflammatory response and prevents apoptosis in peripheral blood cells in adults [13]. Nevertheless, studies on the role of HW in diabetes, particularly in elucidating the mechanism of reducing insulin resistance, are scarce. Thus, a systemic evaluation is necessary to reveal the mechanism and efficacy of HW therapy using an appropriate animal model.

This study aimed to investigate the role of HW on insulin, IRs, and SOD levels in streptozotocin (STZ)-induced diabetic rats.

## Materials and Methods

### Ethical approval

The study was approved by Ethics Committee of Poltekkes Kemenkes Malang, Indonesia with reference number EC00974 2020.

### Study period and location

The study was conducted from April to July 2020 in Laboratory of Animal Experimental, Faculty of Mathematics and Natural Sciences, Universitas Negeri Malang, Indonesia.

### HW preparation

Fresh HW was prepared using hydrogen–water electric generator (DR+Water Electric Generator, PT. Altra Multi Sukses). Fresh HW (3 mL) at a concentration of 1 ppm was administered orally and *ad libitum* for 14 days.

### STZ-induced diabetic rat model

A total of 30 male Wistar rats (200±20 g body weight [BW], 10±2 weeks old) were placed individually in standard cages with free access to food and water. The animals were acclimatized for a week. After acclimatization, the rats were fed a standard chow (n=5) and an HFD (n=25) for 30 days. The fat content (34.2%) of HFD was higher than that of the standard chow diet, which was only 3% [9]. After 30 days, the initial fasting blood glucose levels were measured. STZ-induced diabetic rats were obtained through injection of a repeated low dose of STZ (35 mg/kg BW dissolved in 0.1 M citrate buffer [pH 4.5]) intraperitoneally [8]. After 3 days of injections, fasting blood glucose was measured. The rats were considered DM, and the fasting blood glucose levels were >200 mg/dL [14].

### Experimental design

After the HFD-fed, rats were considered DM, the animals were randomly divided into fivegroups (n=5 rats for each group): (1) N: Normal (standard chow diet), (2) DM: Diabetic rats, (3) DM+Met: Diabetic rats+metformin as drug control (45 mg/kg BW), (4) DM+Met+HW: Diabetic rats+metformin (45 mg/kg BW)+HW, and (5) DM+HW: Diabetic rats+HW.

The treatments were administered orally for 14 days. On 15^th^ day, the animals were dissected after using ketamine and xylazine as anesthetic solutions. The blood was drawn by cardiac puncture, and the organs were collected and stored in 10% formalin for further analysis.

### Immunohistochemical (IHC) assay

Expression of insulin and IRs measurement in the adipose and muscle tissues was performed using the IHC staining method. The adipose and skeletal muscle tissues embedded with paraffin were cut into 5-mm thickness. The slides were incubated in an oven at 40°C overnight. Then, they were then stained with an anti-insulin primary antibody (SC-8033, Santa Cruz Biotechnology, USA) or anti-IRs-1 primary antibody (SC-8038, Santa Cruz Biotechnology) in 2% bovine serum albumin (BSA) (1:500) [15]. In addition, they were stained with secondary antibody goat anti-mouse IgG fluorescein isothiocyanate (02-18-06, KPL, USA) or goat anti-mouse IgG tetramethylrhodamine isothiocyanate (ab6768, Abcam, UK) in 2% of BSA (1:1500) [9]. They were washed with phosphate-buffered saline (PBS) (pH 7.4) 3 times, dried, and then observed under a fluorescence microscope (FSX 100, Olympus, Japan). The expression of insulin or IRs was analyzed and presented as intensity/mm^2^ (Int/mm^2^). The intensity for each slide was measured using the Fiji-ImageJ2 software (https://imagej.net/software/fiji/).

### Enzyme-linked immunosorbent assay (ELISA)

The insulin and IR levels were measured using indirect ELISA [9] with some modifications. Briefly, 50 μL coating buffer and 100 μL sample were added to ELISA well plate and incubated for 1 h at 37°C or overnight at 4°C. After washing with PBS-Tween (PBST), 50 μL of anti-insulin primary antibody (SC-8033) or anti-IRs-1 primary antibody (SC-8038) was added to 2% of BSA (1:500) and incubated for 2 h at 37°C. Then, after washing with PBST, 50 μL of secondary antibody IgG conjugate (S3831, Promega, USA) was added to 2% of BSA (1:1000) and incubated for 1 h at 37°C. Streptavidin horseradish peroxidase (50 μL) was added to the assay buffer after washing with PBST and incubated for 1 h at 37°C. After washing the well plate, 90 μL of tetramethylbenzidine peroxidase substrate (Rockland, USA) was added and incubated for 30 min at 37°C. Then, 50 μL of 1N HCl was directly added as a stop solution and then measured at a wavelength of 450 nm. The levels of insulin or IRs were calculated according to the standard curve. The level of SOD was measured using Rat SOD ELISA Kit (BZ-08188610-EA, Bioenzy, Indonesia) according to the manufacturer’s protocols.

### Statistical analysis

The data were expressed as mean±standard deviation of means. The homogeneity and normality of the data were tested first, followed by a one-way analysis of variance. The significance of differences between the groups was calculated using Duncan’s multiple range tests as a *post hoc* test. A p<0.05 was considered significant.

## Results

### The role of HW in improving the level of insulin and IRs

**Figure-1:**
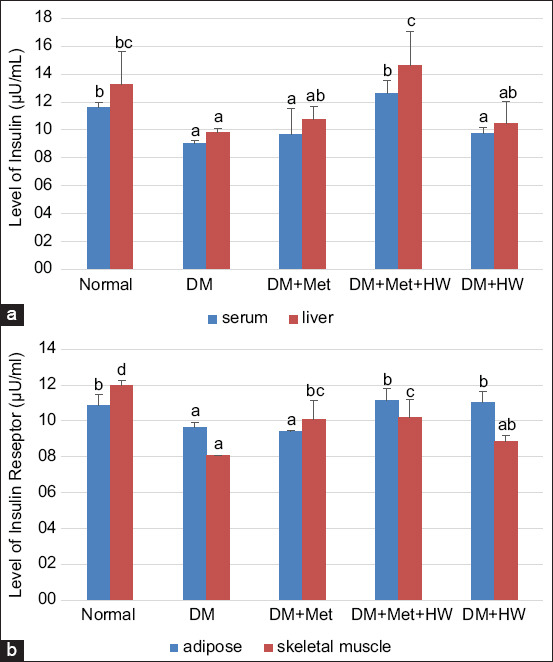
The level of insulin and insulin receptor (IR) in each group. (a) The level of insulin in serum and liver based on enzyme-linked immunosorbent assay (ELISA) method at each group. (b) The level of IR in adipose and skeletal muscle tissue based on ELISA method at each group. The different letters indicate significant differences between groups (p<0.05) according to Duncan’s multiple range test *post hoc* test.

This study demonstrated that STZ-induced diabetic rats exhibited a significant decrease in the level of insulin in the serum and liver compared with normal rats (p<0.05) (Figure-1). However, DM rats showed a significant decrease in IR levels in the adipose and skeletal muscle tissues compared with normal rats. Treatment with HW showed a significant increase in insulin and IR levels (p<0.05). HW also increased the level of insulin in the liver and the level of IRs in the adipose tissue, similar to the normal group. Interestingly, no significant difference was observed between the groups treated with metformin alone and the group treated with HW. These results indicated that HW could improve the level of insulin and IRs in STZ-induced DM rats.

### The role of HW in reducing the expression of insulin and IRs

**Figure-2:**
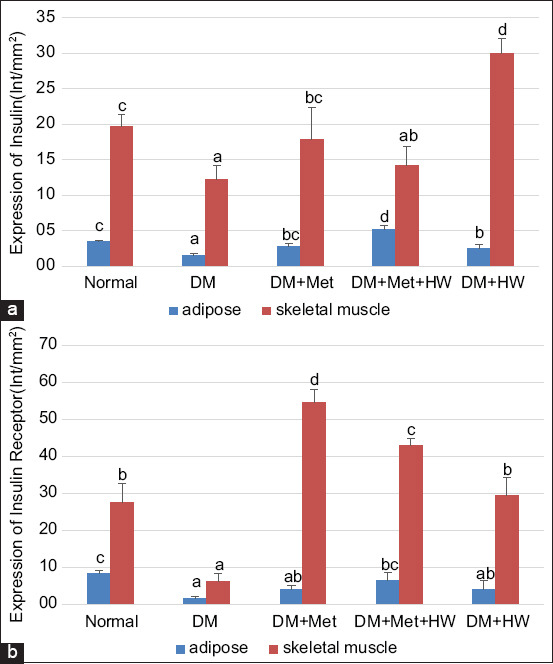
The expression of insulin and insulin receptor (IR) at each group. (a) The expression of insulin in adipose and skeletal muscle tissue based on IHC method. (b) The expression of IR in adipose and skeletal muscle tissue based on immunohistochemical method. The different letters indicate significant differences between groups (p<0.05) according to Duncan’s multiple range test *post hoc* test.

**Figure-3:**
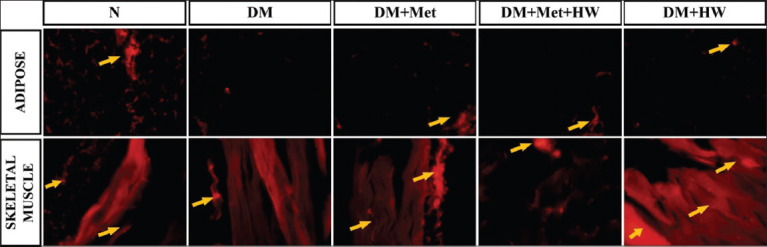
The expression of insulin in adipose and skeletal muscle tissue on each group using the immunohistochemical method. Mag. 400× by fluorescence microscopy with tetramethylrhodamine as secondary fluorescence antibody. The yellow arrow shows the expression of insulin.

**Figure-4:**
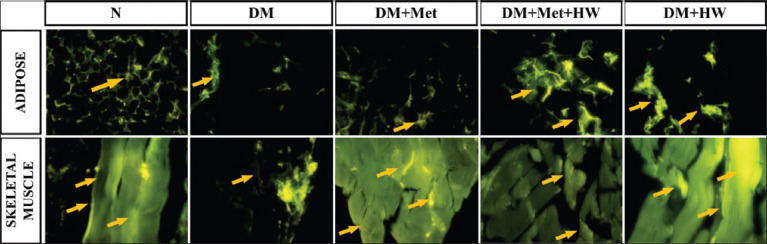
The expression of insulin receptor (IR) in adipose and skeletal muscle tissue on each group using the immunohistochemical method. Mag. 400× by fluorescence microscopy with fluorescein isothiocyanate as secondary fluorescence antibody. The yellow arrow shows the expression of IR.

The expression of insulin and IRs in the adipose and skeletal muscle tissues indicated that the DM rat group has a lower expression of both insulin and IRs than the normal group (p<0.05) (Figure-2). This result indicated that insulin and IRs significantly reduced under the DM condition. HW treatment significantly improved the expression of insulin and IRs in both the adipose and skeletal muscle tissues compared with the DM group (p<0.05) (Figures-3 and 4). Furthermore, no significant difference was observed between treatment with metformin and treatment with HW only, especially in the expression of insulin and insulin receptor substrate in the adipose tissue (Figure-2). HW could be used as a treatment to reduce the expression of insulin and IRs in the adipose and muscle tissues in particular.

### Therapeutic effect of HW in increasing the SOD levels

**Figure-5:**
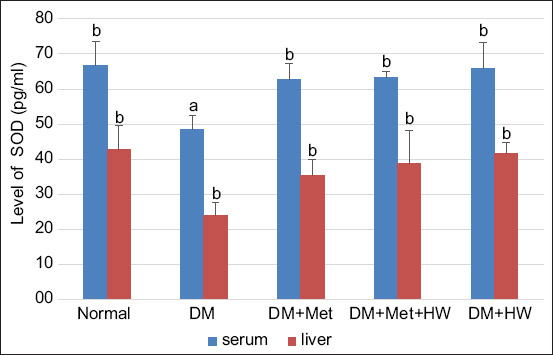
The level of superoxide dismutase in serum and liver based on enzyme-linked immunosorbent assay method at each group. The different letters indicate significant differences between groups (p<0.05) according to Duncan’s multiple range test *post hoc* test.

The results indicated that the DM rat group has the lowest SOD levels in serum and liver. However, the SOD level in the serum and liver significantly increased after treatment with HW (p<0.05) (Figure-5). Interestingly, no significant difference was observed between treatment with metformin and treatment with HW. These results indicate that HW has a therapeutic effect on DM by increasing the level of SOD as an endogenous antioxidant.

## Discussion

DM is a chronic metabolic disease or syndrome characterized by high levels of blood fasting glucose, which becomes a global burden. The incidence of DM is rapidly growing worldwide [16]. The World Health Organization reported that the prevalence of DM over the past few decades has significantly increased. The International Diabetes Federation reported 415 million adults (20-79 years old) with DM in 2015 (8.8%). Meanwhile, the worldwide prevalence is estimated to be 642 million in 2040 (10.4%) [17].

Animal models are widely used to measure and test the efficacy of some treatments, such as the development of some synthetic drugs or herbal medications [18]. STZ is commonly used to induce DM in animal model. STZ exhibits diabetogenic properties, including destroying the pancreatic islet β-cells, which secrete insulin. This destruction causes insulin deficiency and hyperglycemia [19]. However, HFD leads to an imbalance in the lipid uptake, thus affecting the accumulation in nonadipose cells, including the heart, liver, kidneys, skeletal muscle, and pancreas. These mechanisms cause impairment in cellular function [20]. HFD also contributes to the development of insulin resistance in an animal model of impaired glucose tolerance and T2DM [21].

This study indicated that the level and expression of both insulin and IRs in the DM group declined compared with the normal group. The low level and expression of insulin were caused by β-cell destruction due to the STZ stressor. With the higher percentage of β-cell destruction, there will be little insulin production, thus causing hyperglycemia. Okoduwa *et al*. [22] reported that low-dose STZ injection (35 mg/kg BW) in fat-enriched diet rats resulted in a slight decrease in serum insulin levels (14.2%), lower than that in normal diet rats injected with the same dose of STZ. Based on the previous research, the fasting blood glucose levels in DM rats (415.67 mg/dL) increased significantly compared with normal rats (85.60 mg/dL) due to hypoinsulinemia [23].

Furthermore, Szkudelska *et al*. [24] reported that IR expression in the skeletal muscle decreased in Goto-Kakizaki rats with congenital T2DM. The decrease in IR was associated with hyperglycemia due to undelivered signals to the cell, whereas insulin bound to its receptor [25]. However, both stressor STZ and HFD play a role in increasing free radicals. Gofur *et al*. [15] reported that, in DM rats, STZ, and HFD increased the level of malondialdehyde (MDA) as a free radical product. The increase in the free radical product initiated oxidative stress, thus decreasing the level of endogenous antioxidants, such as SOD. Sarkar *et al*. [26] revealed a significant decrease in SOD, GPx, and CAT levels in STZ-induced diabetic rats.

HW has been explored for its biological properties, especially as an antioxidant. A previous study reported by Susatia *et al*. [23] revealed that administration of HW in DM rats has ability to decrease fasting blood glucose significantly compared with DM rats without treatment. Furthermore, HW decreased the level of interleukin 1β (IL-1β) as a pro-inflammatory cytokine and increased the level of IL-10 as an anti-inflammatory cytokine [23]. HFD leads to obesity, which causes pro-inflammatory disease, and administration of HW might suppress the negative effect after consuming HFD by acting as an anti-inflammatory agent [27]. Nevertheless, the results of this study indicated that HW consumption increased the level and expression of both insulin and IRs in DM rats. Administration of HW also increased the level of SOD in the serum and liver. Amitani *et al*. [6] reported that hydrogen promoted glucose uptake into the skeletal muscle due to its antioxidant activity, thus restoring fasting blood glucose levels.

Moreover, HW supplementation can also alleviate oxidative stress MDA and increase serum SOD levels in T2DM [28]. The increase in antioxidant levels prevents cellular damage, especially in the pancreas. The antioxidant activity of HW improved necrosis cells in the islets of Langerhans [23] and increased the number of β-cells that produce insulin.

## Conclusion

Drinking HW is effective in the treatment of DM, especially T2DM. HW with its antioxidant activity improves insulin, IR, and SOD levels in DM rats. No significant difference was observed between the administration of HW and metformin. These findings indicate that HW can be a candidate for the treatment of DM. However, the limitation of this study is that only the expression of protein level was measured; the expression of gene-level was not explored. It should be of future interest.

## Authors’ Contributions

ER and BS: Designed and conceived the study. ER, SNA, and SRL: Performed laboratory tests and data analysis. ER, BS, SNA, and SRL: Drafted and revised the manuscript. All authors read and approved the final manuscript.
